# Chemical stabilization of dispersed *Escherichia coli* for enhanced recovery with a handheld electroflotation system and detection by Loop-mediated Isothermal AMPlification

**DOI:** 10.1371/journal.pone.0244956

**Published:** 2021-01-05

**Authors:** Lena Diaz, Yong Li, Daniel M. Jenkins

**Affiliations:** 1 Department of Molecular Biosciences & Bioengineering, University of Hawaii at Manoa, Honolulu, Hawaii, United States of America; 2 Department of Human Nutrition, Food, and Animal Science, University of Hawaii at Manoa, Honolulu, Hawaii, United States of America; Tallinn University of Technology, ESTONIA

## Abstract

Constraints related to sample preparation are some of the primary obstacles to widespread deployment of molecular diagnostics for rapid detection of trace quantities (≤10^3^ CFU/mL) of food-borne pathogens. In this research, we report a sample preparation method using a novel handheld electroflotation system to concentrate and recover dilute quantities (10^2^−10^3^ CFU/mL) of *Escherichia coli (E*. *coli) 25922* in artificially contaminated samples for reliable, rapid detection by loop-mediated isothermal amplification (LAMP). To protect suspended cells from shear stresses at bubble surfaces, a non-ionic surfactant (Pluronic-F68) and flocculant (chitosan oligosaccharide) were used to aggregate cells and reduce their surface hydrophobicity. Effective conditions for recovery were determined through multifactorial experiments including various concentrations of Pluronic-F68 (0.001, 0.01, 0.1, 1 g L^-1^), chitosan oligosaccharide (0.01, 0.1, 1, 10 g L^-1^), bacteria (10^2^, 10^3^, 10^4^ CFU/mL *E*. *coli* 25922), recovery times (10, 15 and 20 minutes), and degrees of turbulent gas flux (“high” and “low”). The automated electroflotation system was capable of concentrating effectively all of the bacteria from a large sample (380 mL 0.1 M potassium phosphate buffer containing 10^2^ CFU/mL *E*. *coli*) into a 1 mL recovered fraction in less than 30 minutes. This enabled detection of bacterial contaminants within 2 hours of collecting the sample, without a specialized laboratory facility or traditional enrichment methods, with at least a 2–3 order of magnitude improvement in detection limit compared to direct assay with LAMP.

## Introduction

In the past 10 years food safety programs initiated by the United States Food and Drug Administration (FDA) including the Food Safety Modernization Act [1], Current Good Manufacturing Practices (CGMPs) [2] and Hazard Analysis Critical Control Point (HACCP) [3] imply that the next generation of food safety guidance’s need to encompass the entire supply chain, from farm to table. Acknowledging that technological advances introduced a new imperative to ensure food safety the HACCP regulations set forth in 9 CFR Part 417 lifted the uniformed federal control regulations across meat and poultry establishments. The conversion from a one-size-fits-all to the current contemporary conditions that allow establishments to make site-specific production decisions shifted the responsibility of ensuring food safety from the federal inspectors to the industry establishment. This flexible approach permitted establishments to integrate or innovate novel technologies that enable on-site point-of-care (POC) sampling and detection of pathogenic organisms from food and in the environment to control and mitigate the spread of contamination in their unique production processes.

While rapid, portable diagnostic platforms have reached commercial maturity, trace quantities (≤10^3^ CFU) of bacterial contaminants dispersed in ecological scaled sample sizes (hundreds of grams or liters) remain notoriously difficult to detect. Nucleic acid based detection technologies like loop mediated isothermal amplification (LAMP) [4] have significantly shortened the time to detection (30–60 minutes) and are supported in commercial handheld platforms, but typically have a limits of detection (LOD) of 10^3^−10^4^ CFU/mL at best. The implementation of LAMP supporting technologies into routine POC sampling procedures is limited by sample preparation requirements (*i*.*e*. culture enrichment) that produce sufficient target quantities for amplification [5]. Culture enrichment methods can extend the time required to achieve preliminary screening results from 6–8 hours [6] to 3–7 days [7, 8]. Methods like centrifugation, immunomagnetic separation (IMS), and filtration are commonly used to separate or concentrate bacteria cells from sample matrix prior to detection however these technologies are not easily adaptable for point-of-care testing. IMS techniques integrated with 3D-printed microfluidic devices have successfully isolated and separated captured bacteria from unbound magnetic particles for detection of pathogens down to 100 cfu/mL in milk, however IMS itself requires expensive functionalized magnetic particles and external syringe pumps to manipulate the sample [9]. Low cost, simple POC technologies facilitating sample size reduction by concentrating cells into small er volumes while simultaneously recovering all of the initial targeted bacteria prior to the application of nucleic acid amplification could improve sampling techniques required for environmental testing or sites of sporadic contamination.

Production of biodiesel and other natural products from algae requires similar processes such as filtration, coagulation, flocculation, flotation, sedimentation or centrifugation to recover biomass from suspension [10]. Significant research has been conducted into flotation processes for industrial dewatering of algae including by froth flotation [11], dispersed air flotation [12] and, less commonly, electro-flotation [13]. While research on methods to recover viable bacterial cells using flotation is sparse, techniques used in wastewater treatment or industrial algal harvesting involving stabilization and separation of dispersed systems may be applied to separate and concentrate bacteria from dilute suspensions.

Aeration of bioreactors is commonly used to control dissolved gas concentrations in cell culture for production of biological products like therapeutic proteins, vaccines, and antibodies, especially in large industrial systems (> 10,000 L) with high cell densities (>10^6^ CFU/ml) that are highly diffusion limited [14]. Typical aeration processes such as gas sparging, however, can result in significant stress to cells [15], requiring additives to reduce foaming and the shearing stresses due to hydrophobic interactions with cell surfaces [14]. Cell death may also occur in bacteria confined in bubble films when the bubbles rupture [16], or even if the degree of turbulence of circulating media is especially intense resulting in high shear in the liquid phase itself [15, 17].

Turbulent shear stress on cells in a culture can be stabilized by adding surfactants to the media [18]. Surfactants can change interactions between a bubble and surrounding biological material in a fluid by modifying the surface tension forces that typically attract, stress or disperse biomaterial [14, 16, 19]. Pluronic® F-68 is a commercially available non-ionic surfactant that has been widely investigated and shown to protect cells by masking hydrophobic surfaces and thereby reducing shearing effects on the cell membrane from interaction with hydrophobic gas bubbles [20].

Flotation by microbubbles relies on the attachment of a particle to the bubble to form bubble-floc aggregates that rise to the surface of the media. As demonstrated in sewage purification or ore refineries, aggregating particles prior to flotation can result in a substantial increase in particle mass recovered [21]. Considering numerous applications of flotation of biological materials, flotation was optimized to achieve 99% recovery rates of *Chlorella sp*. [22], bacterial suspensions including *Escherichia coli (E*. *coli)* [23, 24], and microalgae [12] by adding cationic polyelectrolytes (*i*.*e*. chitosan) as flocculants to aggregate bacterial suspensions.

Chitosan, characterized as a linear polysaccharide that has various proportions of (1→4) linked 2-acetamido-2-deoxy-β-D-glucopyranose (GlcNAc) and 2-amino-2-deoxy—β-D-glucopyranose [25], is an inexpensive, biodegradable, non-toxic, cationic natural polymer/ polysaccharide obtained by *partial* (~50%) deacetylation of chitin found in the exoskeleton of crustaceans like shrimp [26]. The cationic nature of chitosan is particularly desirable to flocculate and aggregate negatively charged particles. Bacterial cells contain large quantities of side chain amino acids, methyl groups attached to polysaccharides and long chain carbon groups found in lipids; all contributing to the hydrophobicity and predominantly negative surface charge of cell membranes [27]. In gram negative bacterial cells, the anionic phosphate and carboxyl group residing on lipopolysaccharides (LPS) of the outer membrane (OM) can electrostatically interact with the cationic molecules such as the regularly repeating protonated amine groups of chitosan [28]. When used as a flocculant chitosan polyelectrolytes rely on electrostatic interactions to mask the negative charge of cells that would normally disperse stably in a suspension, thereby promoting their aggregation and separation [24].

In this report, we investigate the effects of adding chemical stabilizers (pluronic and chitosan) to enhance concentration, recovery and detection by LAMP of small quantities of dispersed bacterial contaminants by electroflotation (EF) performed in a hand-held automated system [29]. Additionally, a simple sample preparation procedure was developed so that recovered EF samples could be directly added to a LAMP assay without DNA purification or inhibition on LAMP reaction.

## Materials and methods

### Preparation of bacterial cultures, purified DNA and media

*E*. *coli* ATCC strain 25922 bacteria were stored at -80°C prior to experimentation. After removal from storage *E*. *coli* 25922 was propagated twice overnight on plate-count agar (Difco^TM^) at 37°C. Colonies were then transferred into sterilized potassium phosphate buffer (0.1 M, pH 6.6) adjusted to achieve an absorbance of 0.13 at 600 nm as read on a commercially available spectrophotometer (Healthcare Ultraspec^TM^ 10, General Electric, Boston, MA). This absorbance was shown empirically to be equivalent to about 10^8^ CFU/ml (*x* = 1.63 x 10^8^ CFU/mL, s = 2.55x10^7^ CFU/mL, n = 3) through comparison to standard plate counting methods. Bacterial cultures and media were freshly prepared for each electro-flotation experiment. A Wizard genomic DNA purification kit (Promega Corporation, Madison, WI) was used to purify *E*. *coli* 25922 DNA following manufacturer’s protocols.

### LAMP assay design

For detection of *E*. *coli*, we chose to use LAMP, a popular isothermal amplification chemistry that may be especially attractive for use in portable diagnostic systems [30, 31]. To target *E*. *coli 25922* we used a previously characterized LAMP primer set [29] designated EcolC 3109_1 (Table 1; [29]), targeting a conserved glycerate kinase coding region (EcolC 3109, Accession number: CP000946) of generic *E*. *coli* ATCC 8739. All LAMP reactions were performed in 25 μL (total volume) containing 40 pmol of each inner primer (BIP and FIP), 5 pmol of each outer primer (B3 and F3), 20 pmol of each loop primer (LB and LF). Reactions were prepared by adding 5 μL of a stock primer solution and 5 μL of sample to 15 μL of commercially available Isothermal Mastermix with dye (Catalog No. ISO-001, Optigene, Inc., Horsham, UK). All primers were synthesized commercially (Integrated DNA Technologies, Coralville, IA, USA). All reactions were performed in 0.1 mL TempPlate semi-skirt PCR 96-well Plates (Catalog No. 1402–9100, USA Scientific, Inc., Ocala, FL, USA) in a commercial real-time PCR machine (StepOnePlus^TM^ Applied Biosciences, Foster City, CA, USA) incubated at 65°C for 31 minutes. Fluorescence values were recorded every 30 seconds during the 31-minute reactions. The “threshold time” t_T_ was estimated as the amount of time required for the fluorescence value to exceed a threshold value equivalent to the pooled average plus three standard deviations of the fluorescence values observed throughout reactions of triplicate negative control reactions [30]. Reported averages of t_T_ values exclude assays with undefined t_T_ values (t_T_ >31 minutes). Reactions were conducted in triplicate for each template DNA concentration and primer set, including for the non-template controls.

**Table 1:** Loop-mediated isothermal amplification (LAMP) primer sequences.

	Nucleotide Sequence (5' → 3')
Ecol 3109_1 primer set:	Used for specific detection of *Escherichia coli* 25922
Ecol 3109_1 F3	GGCGAATGCCGTTATCCAG
Ecol 3109_1 B3	CGTGACGCTTGAAGTCTGC
Ecol 3109_1 FIP	CGCGCCTGAAAAGCGTAATCC CGCATGACGAATCAGCTCTC
Ecol 3109_1 BIP	CAATCACCGCCGTTTTCCCGT CGATGGGCGAAACAGTGAAT
Ecol 3109_1 LF	TGCTGGCGTCAAGTTTTGG
Ecol 3109_1 LB	CGCCGGTAAGGCCATAAAAA

### Effect of Pluronic and chitosan on LAMP

The addition of chemical additives to any LAMP reaction can affect the assay performance therefore the inhibitory effects of pluronic (Pluronic^®^F-68, non-ionic surfactant, Thermo Fisher Scientific Inc., Waltham, MA, USA) and chitosan (chitosan oligosaccharide RCHOF, molecular weight = 340–1,600 Da, Food Grade, Qingdao BZ Oligo Biotech Co., Ltd, Qingdoa, China) on LAMP were evaluated. Varying concentrations of pluronic (0%, 0.05%, 0.1%, 0.5%, 1.0%) and chitosan (0, 0.01, 0.1, 1, 10 g L^-1^) prepared in sterilized DI water and 0.1 M phosphate buffer (pH 5.8) respectively were added to individual LAMP assays. 25 μL reactions were prepared by adding 5 μL of a stock primer solution (Ecol 3109_1) and 5 μL of sample containing 4 μL of tested concentrations of pluronic or chitosan and 1 μL containing 0.2 ng of purified *E*. *coli 25922* DNA to 15 μL of commercially available Isothermal Mastermix with dye (Catalog No. ISO-001, Optigene, Inc., Horsham, UK). All conditions were tested in triplicate including positive and negative controls.

#### Inhibitor removal

Chitosan binding to anionic DNA can prevent LAMP primer annealing and inhibit amplification. To prevent LAMP inhibition, chitosan can be transformed from a DNA binding state to an insoluble DNA release state by adjusting the sample media pH (5.8) above the pKa (~9.5) of the amino groups in chitosan. To test if changing the pH of chitosan containing samples could release DNA from chitosan and prevent LAMP inhibition, sodium hydroxide was added to chitosan containing samples. Simulated EF samples (0.1 M potassium phosphate buffer pH 5.8) were prepared containing 0.1 g L^-1^ pluronic and varying concentrations of chitosan (0.01, 0.1, 1, 10 g L^-1^). The final volume of each sample was 1 mL. 4 μL of a stock (10 ng/μL) purified *E*. *coli 25922* DNA was added to each 1mL aliquots containing chitosan + pluronic + phosphate buffer and mixtures allowed to sit for 10 minutes. The final concentration of *E*. *coli* DNA in each 1 mL sample was 0.04 ng/μL. Next, the pH of was adjusted from pH 5.8 to approximately pH 11 by adding 99 μL of 1 M NaOH to each 1 mL simulated EF sample and allowed to stand at room temperature for 10 minutes. Subsequently pH adjusted samples were vortexed at medium speed for 10 seconds, then centrifuged at 1300 rcf for 3 minutes (Eppendorf centrifuge 5415D, Hamburg, Germany). Individual 25 μL LAMP assays were prepared by pipetting 5 μL of the supernatant from each sample as described above to 15 μL Isothermal Mastermix and 5 μL of a stock primer solution (Ecol 3109_1) in a 0.1 mL LAMP reaction tube. Each LAMP reaction contained a final *E*. *coli* DNA amount of 0.2 ng. For each tested chitosan concentration (4 concentrations total) a corresponding duplicate control sample was made except without the addition of sodium hydroxide. The pH was measured at each step of the above procedure prior to running a LAMP reaction using an AB15 Plus meter (Accumet Basic, Fisher Scientific). Three experimental LAMP assay replicates were performed for each sample composition to determine the effects on LAMP threshold times (t_T_). Negative and positive controls were prepared using the same method as previously described except negative controls did not contain any template DNA. Both positive and negative controls contained pluronic but not chitosan in phosphate buffer pH 5.8 adjusted to pH 11 with 1 M NaOH.

### Preparation of spiked electroflotation samples

380 mL of sterile phosphate buffer (0.1 M, pH 5.8) was inoculated with appropriate volumes of freshly prepared *E*. *coli* 25922 culture in 500 mL sterilized flasks to achieve 10^2^−10^4^ CFU/mL bacterial suspension concentrations. To homogenously disperse bacteria into suspension, samples were mechanically shaken briefly (90 seconds) after inoculation and used promptly for subsequent electro-flotation experiments.

### Electroflotation system control

Flotation of *E*. *coli* 25922 was carried out in a custom engineered handheld electroflotation (EF) cell with an approximate 400 mL capacity [29]. Electrolysis reactions were supported on inert platinum plated titanium electrode arrays. A graphical interface allows the user to define process parameters including durations (min.), voltage (3.5–12 V) or current (0–1000 mA), frequency (0–100 Hz), and duty cycle (1–100%) applied to the electrode arrays. The entire process consists of two steps, 1) concentration and 2) recovery, allowing complete automation of the sample preparation process. During the concentration step, collimated microbubbles from an inner electrode array direct particulates into an inverted conical collection area in the lid of the cartridge. To eject concentrated particulates in the recovery step, a concentric electrode array is energized to force gas into a trap, displacing sample from a port at the top of the collection area.

### Preliminary electroflotation of *E*. *coli 25922*

Prepared media samples at 27°C were gently poured into the electro-flotation chamber and sealed with the lid. Previous studies on electroflotation have demonstrated that the volumetric rate of bubbles passing through a cross sectional area at any given time (flux rate) increase with higher levels of current density applied across the electrode surfaces [32–34]. Furthermore, high frequencies and large duty cycle ratios can increase fluid circulation, turbulent mixing and stirring [35]. To investigate the effect of excessive mixing during flotation due to the “stirring effect” samples were subjected to “high” turbulence (HT) (500mA/ 100 Hz/ 75% duty cycle for concentration, 650 mA/ 100 Hz/ 75% duty cycle for recovery) or “low” turbulence (LT) (300mA/ 20 Hz/ 30% for concentration and 600mA/20 Hz/ 50% duty cycle for recovery), for durations of 10, 15 or 20 minutes, and for each bacterial concentration (10^2^, 10^3^, or 10^4^ CFU/mL). Three replicates were performed for each experimental treatment without additional chemical additives.

### EF treatments with pluronic F-68

To enhance viable cell recovery, 0.0038, 0.038, 0.380 and 3.8 mL of pluronic (10%) was added to 380 mL of inoculated EF samples containing 10^2^ and 10^3^ CFU/mL of *E*. *coli* 29522 to achieve pluronic concentrations of 0.001, 0.01, 0.1, 1 g L^-1^ respectively and subjected to 15 min HT EF and 20 min LT EF. Three experimental replicates were performed for each varying pluronic concentration condition.

### EF treatment with chitosan and pluronic

To aggregate cells into shear protected flocs, agricultural grade chitosan oligosaccharide (soluble in pH 5–7) was added to spiked EF samples containing 10^2^ CFU/mL of *E*. *coli* 29522 to achieve final concentrations of 0.001, 0.01, 0.1, 1 g L^-1^. Appropriate chitosan concentrations were prepared by serially diluting a stock concentration of 10 g L^-1^ chitosan. Pluronic was added in the same way as previously described. Next, the cultures were placed on a shaker for 30 minutes at 50 rpm and then gently transferred to the EF cartridge and subjected to 20 min LT of EF treatment. Three experimental replicates were performed for each varying chitosan concentration condition.

### Recovery of electroflotation treated samples and DNA template extraction

The first 3 mL displaced from every EF treatment condition were collected into individual 1 mL fractions in Eppendorf tubes. DNA from all recovered fractions was extracted by boiling (crude cell lysate method; 100°C for 10 min [36]) followed by 15 seconds of low speed vortexing. For detection of *E*. *coli 25922* in recovered fractions from EF treated samples, 5 μL of crude lysed sample was directly used in an individual LAMP assay. For every 1 mL fraction recovered, 3 LAMP assays were performed. Samples containing chitosan were processed in the same way except the pH of 5.8 was adjusted to approximately pH 11 with sodium hydroxide solution following crude cell lysis. This method is described in further detail in the above section “Inhibitor removal”. Three experimental replicates were performed for each treatment condition.

### Statistical analysis

To evaluate the inhibition effects of pluronic and chitosan on a LAMP assay, differences in threshold times were evaluated by linear regression or one-way ANOVA. Dunnett’s *a posteriori* analysis was used to identify specific concentrations of chitosan that demonstrated significant inhibition on LAMP threshold times.

The performance of the electroflotation system is evaluated by effects on LAMP detection rates (0–100%) and the threshold times (t_T_) (i.e. time to detection) of *E*. *coli* 29522 from samples subjected to various EF treatments containing chemical additives (pluronic, chitosan) in comparison to EF treatment control samples without chemical additives. Differences in LAMP threshold times (t_T_) or LAMP detection rates were evaluated using one-way or two-way ANOVA. Tukey’s multiple comparisons *a posteriori* analysis was used to identify specific experimental treatment conditions (*i*.*e*. simple main effects) that were different than corresponding controls. The Tukey’s test compared the mean detection rates (n = 27 LAMP assays) from each treatment condition within a specific tested bacterial concentration (CFU/mL).

Positive detection was classified for threshold times values t_T_<28 minutes. Averaged threshold times exclude t_T_ values (t_T_ >31 minutes). Significance was imputed for p-values less than 0.05.

## Results

### Pluronic F-68 inhibition on LAMP

**Fig 1:**
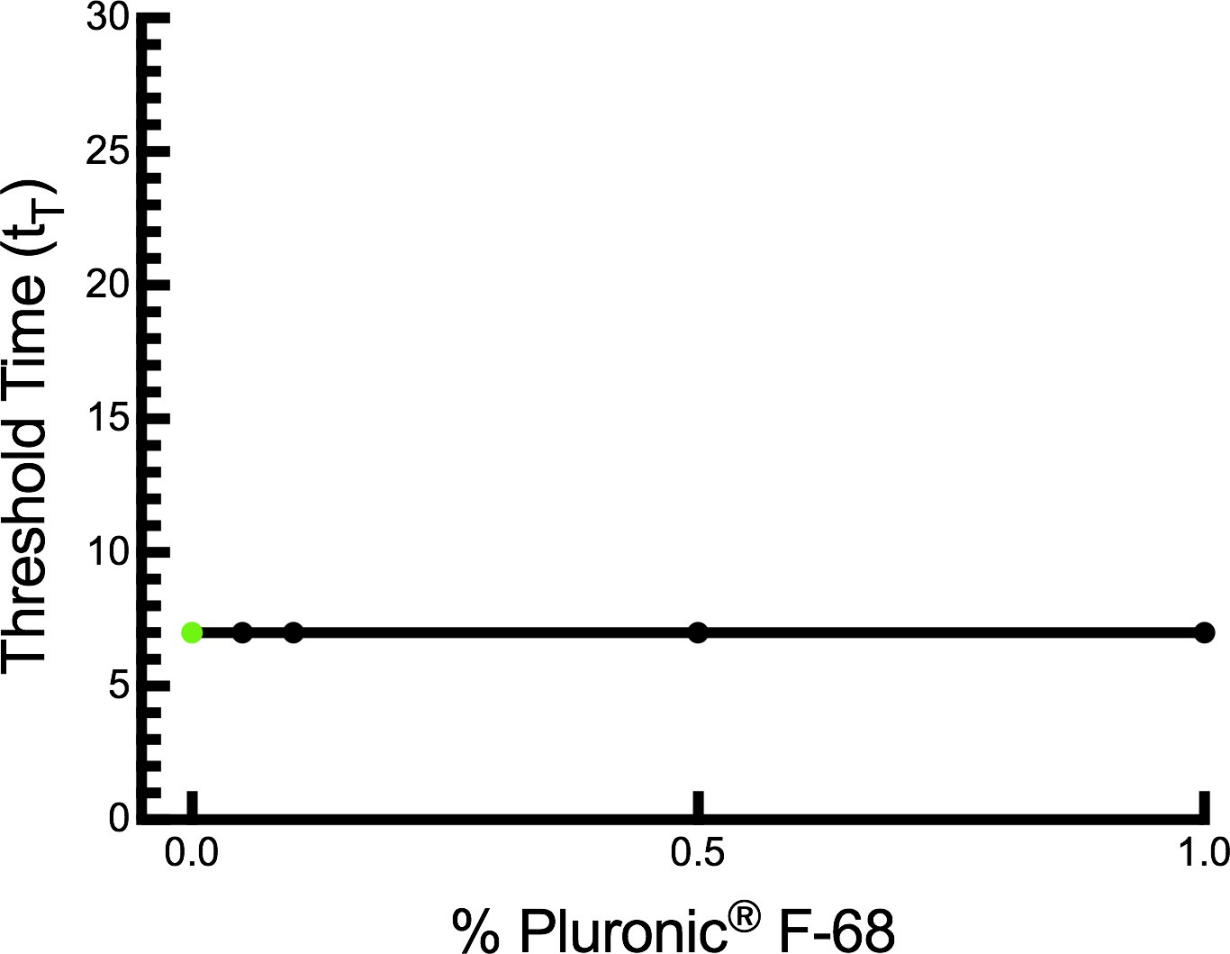
Inhibition on LAMP assays by pluronic. Observed LAMP threshold times for samples containing varying concentrations of pluronic (0.0%, 0.05%, 0.1%, 0.5%, 1.0%). Control group (0% pluronic) indicated by green dot. Each data point represents 3 replicates at the given condition.

LAMP assays were not inhibited by the addition of pluronic to samples at any of the tested concentrations (0.0%, 0.05%, 0.1%, 0.5%, and 1.0%) (Fig 1). Inhibition on LAMP, characterized by increased threshold times, was evaluated by linear regression. The threshold time (t_T_) for all trials at every tested concentration of pluronic was 7 minutes, such that the slope (effect of pluronic concentration on t_T_) was equal to 0 (Y = 7.0, R^2^ = 1) and no inhibition on LAMP was observed.

### Chitosan inhibition on LAMP

**Fig 2:**
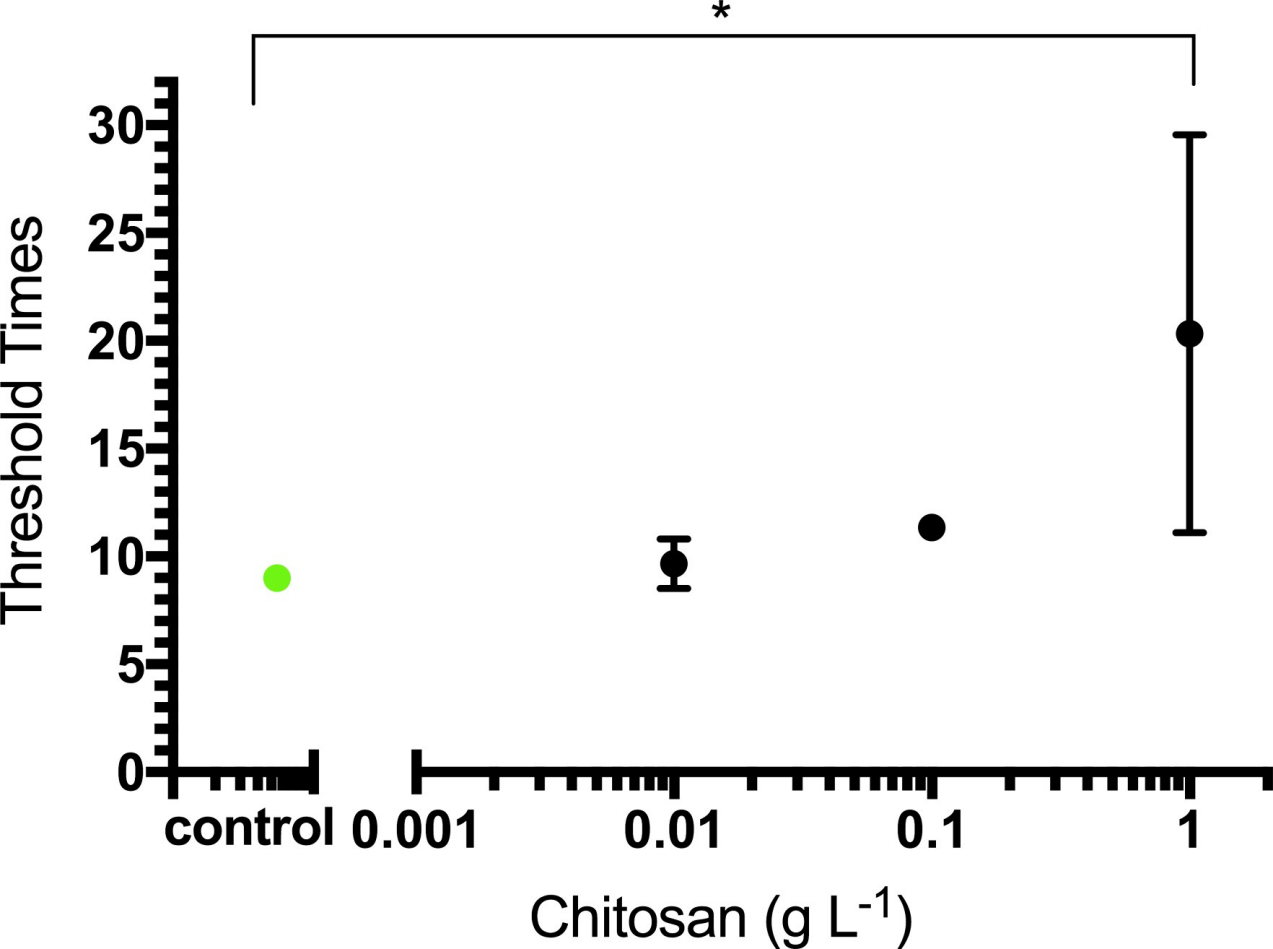
Inhibition on LAMP assays by chitosan. Observed LAMP threshold times for EF samples containing varying concentrations of chitosan (0, 0.01, 0.1, 1 g L^-1^). Chitosan completely inhibited LAMP at concentrations above 1 g L^-1^. Each data point represents the mean threshold time (t_T_) from (n = 3) LAMP assays. Treatments significantly different than control (0 g L^-1^ chitosan, green dot) are indicated by asterisk (* p<0.05) Error bars are standard errors of the mean.

Significant effects on threshold times (t_T_) of varying chitosan concentrations were observed (p = 0.0004). Complete inhibition of LAMP occurred from samples containing chitosan concentrations above 1 g L^-1^. This resulted in no detection of *E*. *coli 25922* by LAMP under these conditions. Using Dunnett’s multiple comparison *a posteriori* analysis, chitosan concentrations of 1g L^-1^ resulted in significant differences, but not complete inhibition, in mean threshold times compared to the control with no chitosan (Fig 2).

### Preventing LAMP inhibition by chitosan

**Fig 3:**
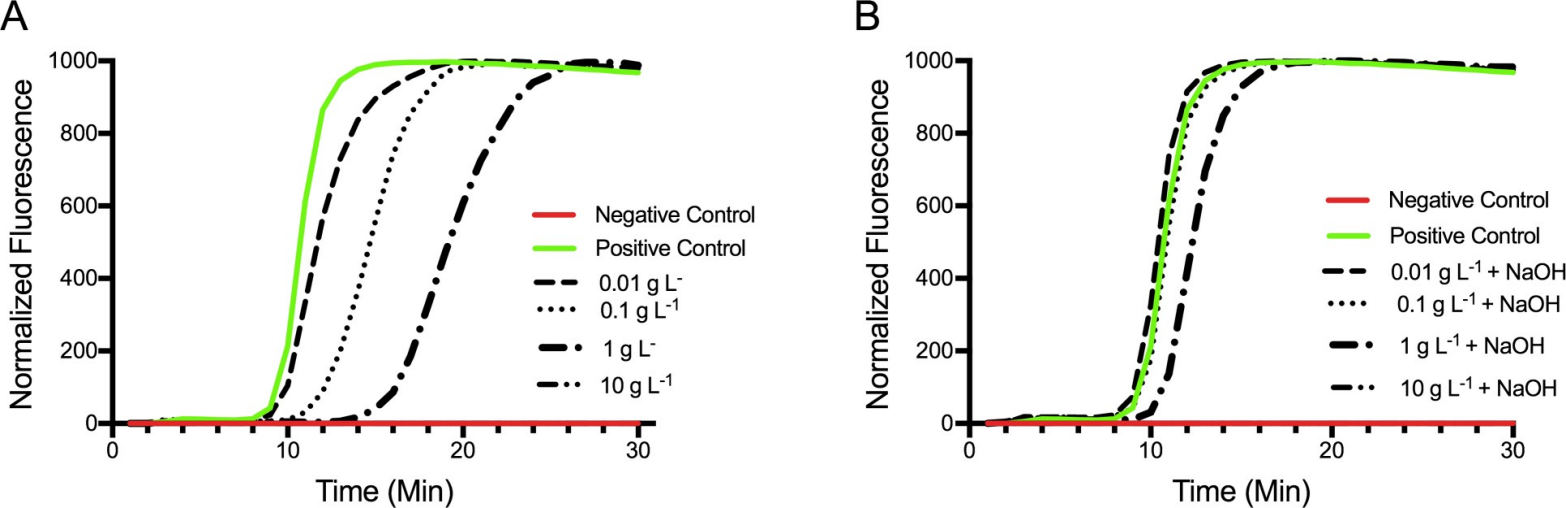
Increasing sample pH to prevent LAMP inhibition by chitosan. Representative LAMP amplification curves for samples (pH 5.8 and pH 11) containing 0.0, 0.01, 0.1 and 1 g L^-1^ chitosan. Samples (pH 5.8) containing 10 g L^-1^ chitosan completely inhibited LAMP. Samples containing 1 g L^-1^ chitosan significantly inhibited LAMP amplification while samples containing 0.01 and 0.1 g L^-1^ only slightly inhibited LAMP when compared to the positive control not containing any chitosan (A). The addition of NaOH to samples removed most of the inhibition on LAMP (B). All reactions contained 0.2 ng *E*. *coli 25922* DNA except the negative control. 3 replicate assays were performed for each condition. One representative curve from each treatment condition is shown.

The amino group of chitosan is cationic below its pKa (~pH 9.5). In this state, it will bind through electrostatic interaction to negatively charged particles (i.e. *E*. *coli* and anionic DNA) interfering with LAMP primer annealing and therefore inhibiting amplification. Samples containing 1 g L^-1^chitosan significantly (p = 0.025) inhibited LAMP amplification increasing the time to detection by 6 minutes (*t_T_* = 15, σ = 9.24) compared to the control samples (*t_T_* = 9, σ = 0) (Fig 3A) and resulting in marked decrease in reaction consistency. While samples containing 0.01 g L^-1^ (p = 0.998) and 0.1 g L^-1^ (p = 0.897) chitosan were not shown to have statistically significant changes in t_T_ relative to controls, this was largely due to reduced reaction consistency under these conditions, and average threshold times in fact increased from 9 min for the control to 9.6 and 11.33 min for the two respective treatments (Fig 3A). Treatment with sodium hydroxide decreased the inhibition on LAMP amplification in samples containing lower concentrations of chitosan (0.01–0.1 g L^-1^) and also in samples containing high concentrations of chitosan (1 g L^-1^) so that amplification was normalized compared to control samples at the equivalent template DNA concentration (Fig 3B). LAMP amplification was inhibited completely in samples containing chitosan in concentrations greater than 1 g L^-1^ and sodium hydroxide was ineffective to improve amplification (Fig 3B). Electroflotation treatments will non-specifically concentrate any particle ranging in size from 0.5 microns to 200 microns, including chitosan aggregates. Although the concentrations of chitosan used as a flocculant in this research (i.e. 0.01 and 0.1 g L^-1^) were below levels causing complete inhibition it is important to note that the observed slight inhibition on LAMP will increase as the concentration of chitosan increases in the recovered samples.

The addition of NaOH solution with a pH of 13.694 to the EF samples adjusted the pH value of the sample mixture to approximately 11 (Table 2). This value is well above the pKa (~pH 9.5) of the amine groups on chitosan. The chitosan used in this study is water soluble (~pH 7–8) and precipitated out of solution at pH 11. The precipitated chitosan was collected at the bottom of the tube by brief centrifugation and the pH adjusted EF sample was pipetted from the top of the supernatant and added directly to the LAMP assay reaction mix. Once the sample is added to the LAMP reaction tube, the buffering capacity of the isothermal mastermix, likely containing Tris-HCl or equivalent, adjusted the reaction mix with EF sample pH to 9. At pH 9 chitosan is in a cationic DNA binding state (i.e. below the pKa). However, at this point in the sample preparation procedure most of the chitosan was removed, or present in trace quantities, from the sample so that inhibition on LAMP amplification was adequately reduced.

**Table 2:** Sample media pH values (n = 3) for each stage during sample preparation and LAMP assays.

Sample type	1 M NaOH solution	EF sample	NaOH treated EF sample	LAMP reaction mix	Reaction mix w/ EF sample
*Mean pH*	13.694	5.848	11.064	8.5	9
*SD*	0.184	0.008	0.005	0	0

SD, standard deviation; M, molar; EF, electroflotation; LAMP, Loop-Mediated Isothermal AMPlification

### EF treatment with pluronic

To protect cells from lysis by hydrodynamic shear forces during EF treatments, varying concentrations of pluronic (0.001, 0.01, 0.1, 1.0 g L^-1^) were added to EF samples. In preliminary experiments 0.001 g L^-1^ pluronic concentration was presumed too low to affect EF treatments and did not significantly change the detection rates by LAMP. On the other hand, addition of 1 g L^-1^ pluronic to EF treatments resulted in undesirable amounts of foam formation during electrolysis, leading to premature sample displacement and potential aerosolization of the target pathogen. Therefore, after preliminary screening of pluronic concentrations, 0.1 and 0.01 g L^-1^ were used for more detailed experiments.

**Fig 4:**
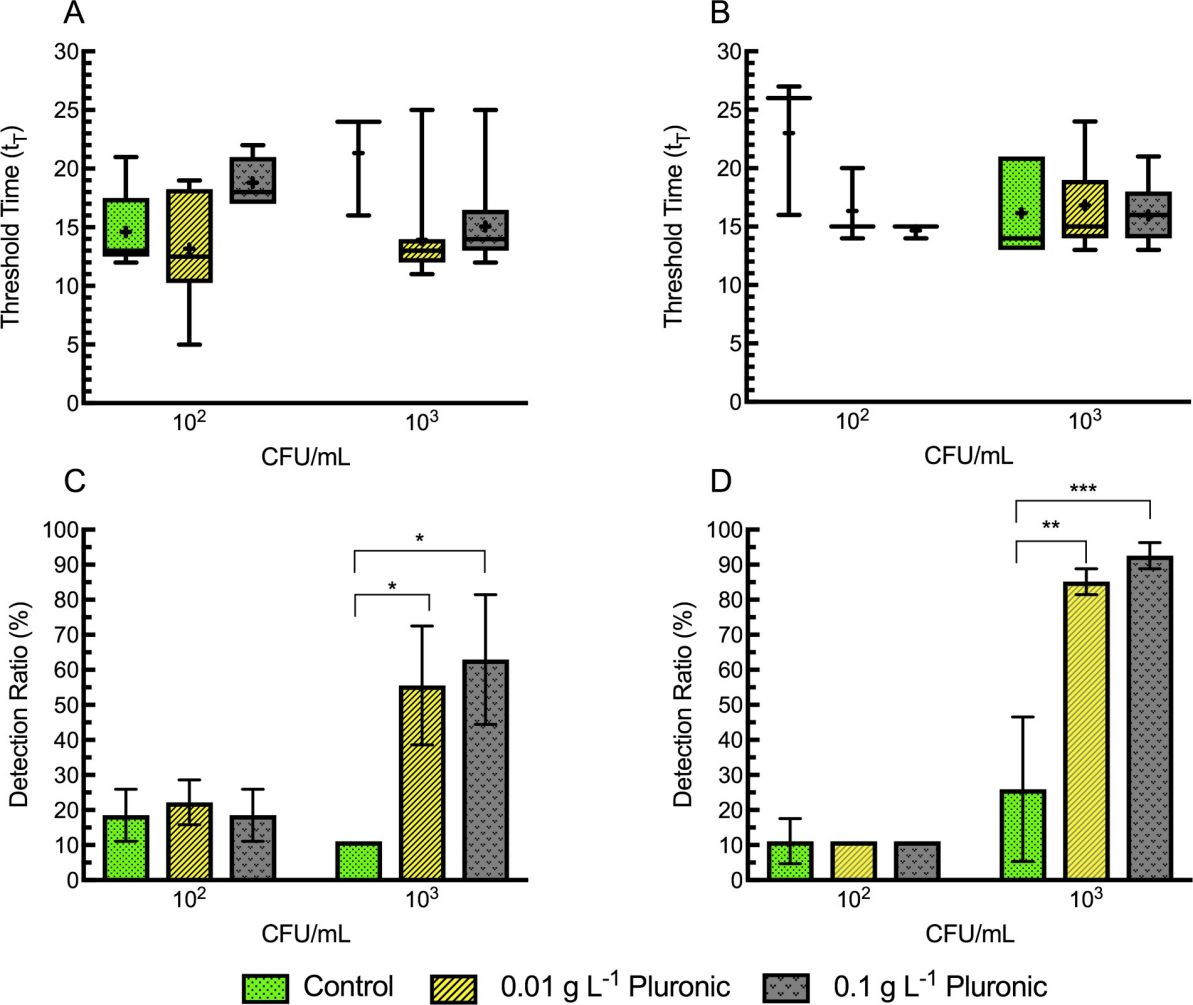
LAMP assay sensitivity of electroflotation treated samples with pluronic F-68. LAMP threshold times (t_T_) after 15 min high turbulence (A) or 20 min low turbulence (B) EF with the addition of 0.0 (control), 0.01 and 0.1 g L^-1^ pluronic to EF treatments. In (C; high turbulence) and (D; low turbulence), each bar represents the mean detection ratio from 27 assays conducted on samples from 3 replicated EF treatments. (9 assays/ treatment, n = 3 for each treatment). Treatments significantly different than controls are designated with asterisks (*p<0.05, **p<0.01, ***p<0.001). In (C) and (D) error bars are standard errors of the means. For (A) and (B), whiskers are from min to max and means are indicated by +. The box extends from the 25th to 75th percentiles.

No significant differences in LAMP threshold times (t_T_) were observed for either 15 min HT-EF (Fig 4A) or 20 min LT-EF (Fig 4B) treatment at any tested bacterial concentration (10^2^, 10^3^ CFU/mL). With respect to LAMP detection rates, analysis of 15 min HT-EF treated samples using a two-factor ANOVA showed a significant effect of bacterial concentration but no significant effect of pluronic concentration or interaction between these factors (Table 3). A subsequent *a posteriori* analysis compared the mean detection rates (n = 27 LAMP assays) from each treatment condition within a given bacterial concentration to every other mean (Table 4). This test identified 0.01 g L^-1^ and 0.1 g L^-1^ pluronic concentrations tested at 15 min HT-EF with 10^3^ CFU/mL had significantly higher rates of detection rates when compared to corresponding controls without pluronic (10%) (Fig 4C). In contrast, a two-factor ANOVA analysis of 20 min LT-EF treated samples showed there was significant effect of bacterial concentration, pluronic concentration and interaction between these factors on LAMP detection rates (Table 3). Subsequent *a posteriori* analysis (Table 4) identified 0.01 L^-1^ and 0.1 g L^-1^ pluronic concentrations tested at 10^3^ CFU/mL had significantly different detection rates of 85.18% and 92.59% respectively when compared to corresponding controls without pluronic (25.9%) (Fig 4D). This is a significant improvement from the ~25% detection rates that were observed in corresponding controls without pluronic and 0% detection rates without electroflotation [29].

**Table 3:** Results of two-way ANOVA analyzing the effects of initial bacterial inoculum and pluronic concentrations on detection rates (n = 27 LAMP assays) of *E*. *coli 25922*.

Factor	15 min. HT-EF			20 min. LT-EF		
	^*‡*^*% of total variation*	*p-value*	^*†*^*p-value summary*	^*‡*^*% of total variation*	*p-value*	^*†*^*p-value summary*
*Interaction*	18.77	0.0965	ns	15.72	0.0059	**
*Bacterial concentration*	20.78	0.0271	*	56.94	p < 0.0001	****
*Pluronic concentration*	21.07	0.0764	ns	15.72	0.0059	**

*p<0.05

**p<0.01; ***p<0.001

****p<0.0001; ns, non-significant p-value; alpha = 0.05; HT-EF, high turbulence electroflotation; LT-EF, low-turbulence electroflotation.

^*†*^Treatment conditions that have a statistically significant effects are designated with asterisks.

^*‡*^The percentage of variation represents the variability due to interaction between bacterial and pluronic concentrations, the percentage due to bacterial concentration and the percentage due to pluronic concentration. The remainder of variation is among replicates.

**Table 4:** Tukey’s *a posteriori* test identifies specific experimental treatment conditions that were different than corresponding controls.

Bacterial Concentration	Treatment conditions	Comparisons (Condition A vs Condition B)		p-value	^†^p-value summary
		^‡^Condition A	Condition B		
*10^2^ CFU/mL*	15 min HT-EF	Control	0.01 g L^-^ Pluronic	0.9715	ns
		Control	0.1 g L^-^ Pluronic	>0.9999	ns
		0.01 g L^-^ Pluronic	0.1 g L^-^ Pluronic	0.9715	ns
	20 min LT-EF	Control	0.01 g L^-^ Pluronic	>0.9999	ns
		Control	0.1 g L^-^ Pluronic	>0.9999	ns
		0.01 g L^-^ Pluronic	0.1 g L^-^ Pluronic	>0.9999	ns
*10^3^ CFU/mL*	15 min HT-EF	Control	0.01 g L^-^ Pluronic	0.043	*
		Control	0.1 g L^-^ Pluronic	0.019	*
		0.01 g L^-^ Pluronic	0.1 g L^-^ Pluronic	0.8914	ns
	20 min	Control	0.01 g L^-^ Pluronic	0.0016	**
		Control	0.1 g L^-^ Pluronic	0.0006	***
	LT-EF				
		0.01 g L^-^ Pluronic	0.1 g L^-^ Pluronic	0.8344	ns

^‡^ Control groups did not contain any pluronic.

*p<0.05

**p<0.01

***p<0.001; ns, non-significant p-value

^†^ Specific experimental treatment conditions (*i*.*e*. simple main effects) that were different than corresponding controls are designated with asterisks.

Low turbulence conditions had overall greater increased detection rates by LAMP when the sample media was supplemented with a higher dosage (0.1 g L^-1^) of pluronic. Therefore in subsequent experiments, to investigate the effects of chitosan, EF treatments were conducted only on samples containing 10^2^ CFU/mL and 0.1 g L^-1^ pluronic at 20-minute LT EF conditions.

**Fig 5:**
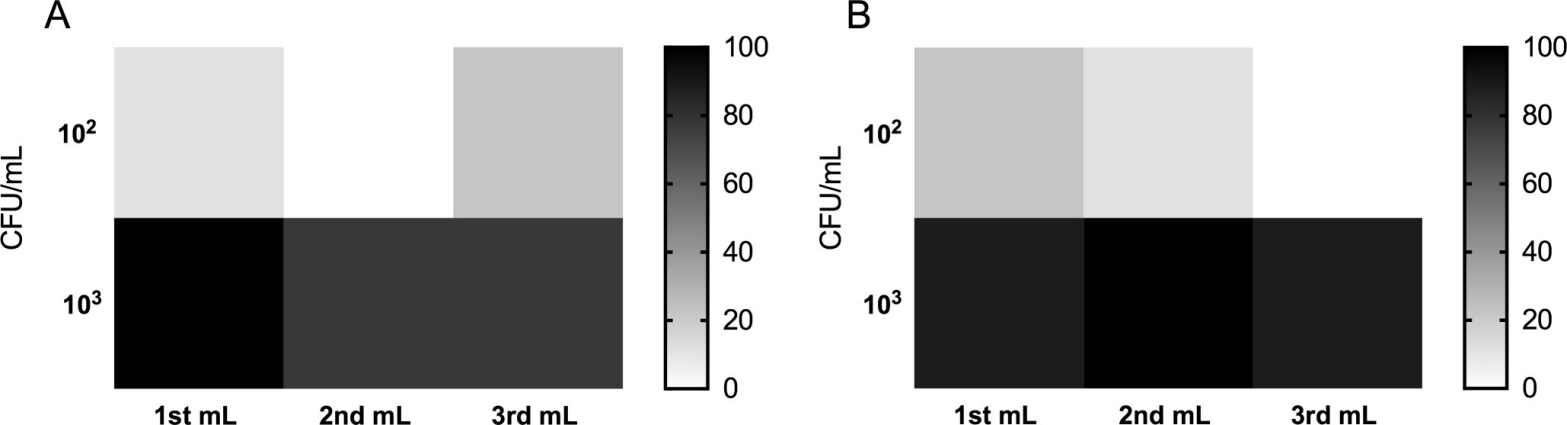
Partitioning of detectable cellular material in the first three recovered 1 mL fractions from EF with pluronic. Heat maps show percentages of LAMP assays positively detecting *E*. *coli 29522* in each recovered 1 mL fraction for the different conditions. Each square shows the mean detection rate of 9 LAMP assays testing recovered fractions containing 0.01 g L^-1^ pluronic (A) and 0.1 g L^-1^ pluronic (B) treated with low-turbulence electroflotation conditions.

To qualitatively assess the distribution of *E*. *coli* in the effluent recovered from EF treatments, the first 1 mL fractions recovered from 3 separate EF experiments at each condition were assayed by LAMP in triplicate. Differences in recovered quantities of *E*. *coli* were then inferred from increased detection rates and/or diminished threshold times. In half of the conditions tested (0.01 g L^-1^ pluronic and 10^3^ CFU/mL, and; 0.1 g L^-1^ pluronic and 10^3^ CFU/mL) detectable DNA was more likely to be concentrated into the 1^st^ mL fraction, though distributions in other conditions were more random (Fig 5A). These results are generally consistent with the mechanism of cell protection with pluronic, whereby cells are more likely to remain intact and in stable suspension, so that the partitioning effect of momentum transfer from bubbles results in a gradient of higher concentration in the conical collection chamber, but not complete aggregation of cellular material at the surface.

In contrast samples containing 0.1 g L^-1^ (Fig 5B) pluronic yielded more positive LAMP assays in the 2^nd^ mL than the 1^st^ mL. For samples containing 10^2^ CFU/mL *E*. *coli* there was an even more variable distribution pattern of quantifiable DNA as many of the recovered samples were below the detection limit of the LAMP assay. The fractions collected for samples containing 10^3^ CFU/mL did contain higher concentrations of DNA (by one order of magnitude) when compared to corresponding controls however the results were inconsistent and there was as much recovered in the third fraction as the first. While there is a definite density gradient established by the flotation process, turbulent mixing of the effluent likely prevents all the particulates from concentrating at the very top of the electroflotation chamber column.

### EF treatment with chitosan and pluronic

**Fig 6:**
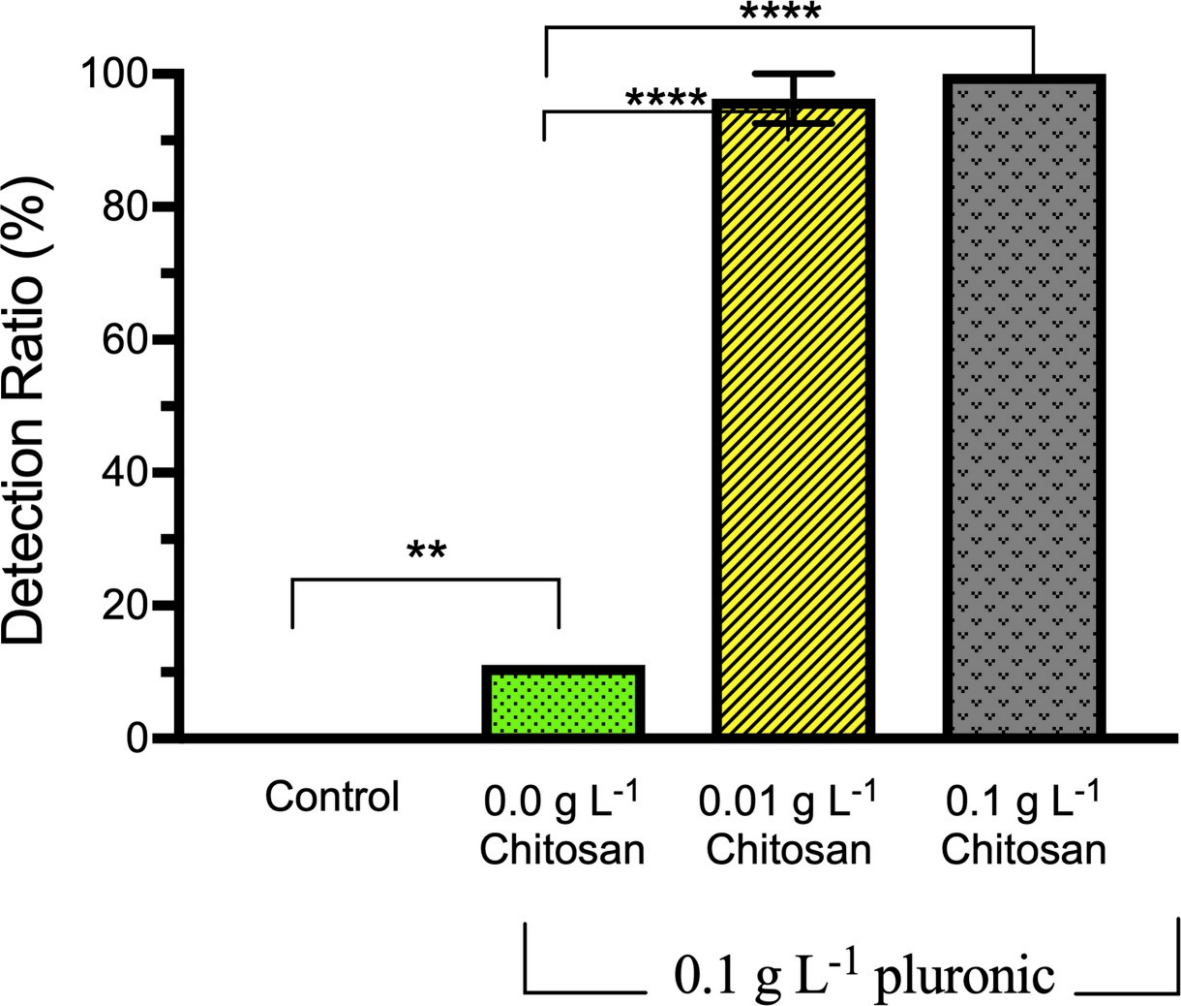
LAMP assay detection rate of electroflotation treated samples with chitosan and pluronic. 10^2^ CFU/mL, Low turbulence, 20-minute treatment with the additions of 0.1 g L^-1^ pluronic + 0.01 g L^-1^ chitosan. Control contained 0.1 g L^-1^ pluronic but no chitosan. Each bar represents the total detection rate from 9 assays testing only the 1^st^ mL collected from 3 replicated EF treatments (3 assays/ 1 mL, n = 3). Treatments significantly different than controls are designated with asterisk (**p<0.01, ****p<0.0001). Error bars are standard errors of the means.

Following 20-minutes of low turbulence EF treatment of media with 10^2^ CFU/mL *E*. *coli* 25922 and 0.1 g L^-1^ pluronic, LAMP assays positively detected the bacteria with mean detection rates of 100% and 96.3% respectively in samples with 0.1 or 0.01 g L^-1^ chitosan. Statistically significant differences (p = 0.0001) in detection rates were observed for all treatments containing chitosan (0.1 or 0.01 g L^-1^) when compared to the corresponding control sample with pluronic but no chitosan and detection rate of 10% (Fig 6). These results were confirmed using Dunnett’s multiple comparison *a posteriori* test.

## Discussion

The degree to which the EF system was capable of concentrating bacteria dispersed in media was measured indirectly by observing changes in detection rate of LAMP assay. The LAMP assay could detect dispersed *E*. *coli* present in quantities of 10^4^ CFU/mL and 10^5^ CFU/mL at rates of ~50% and 100% respectively [29]. Optimizing surfactant (Pluronic® F-68) and flocculant (chitosan) concentrations eventually allowed us to detect bacterial quantities of 10^2^ CFU/mL at average rates of 96.3%-100%. By interpolating these detection rates into the LAMP LOD standard curve suggest that the recovered 1 mL fraction is functionally equivalent to samples containing 10^4^−10^5^ CFU/mL and that all or nearly all of the bacteria from the original 380 ml media sample containing 10^2^ CFU/mL was recovered.

In one subsequent experimentation trial conducted after this study the user was unable to detect *E*. *coli* in any of the collected fractions from a sample originally containing 10^2^ CFU/mL, chitosan and pluronic. While there are numerous reasons for these results including the use of different lot batches of LAMP reagents, different bacterial stocks, etc., the inconsistencies in recovery rates are more likely attributed to a few compounded factors. Firstly, the current prototype of the electroflotation device is custom made using machined or hand cut parts and is cumbersome to assemble and load the sample. Any slight deveation from the standard optimized protocol when loading the sample can affect where the sample is contained in the flotation chamber, the time it takes the sample to process (i.e. eject from the cartridge), the concentration efficiency and therefore downstream detection of the recovered material. It is possible that some of the flocculated bacterial aggregates concentrate in the gas trap instead of the conical collection area preventing those trapped bacteria from being recovered. Secondly, turbulent mixing of the effluent prevents all of the particulates from concentrating at the very top of the column. Depending on the quantity of bacteria trapped and unable to concentrate in the intended chamber, this would significantly lower the rate of detection, especially concerning for lower quantities of bacterial concentrations (i.e. 10^2^ CFU/mL). In the future design modifications to the electroflotation system that simplify assembly (i.e. screw on cap and embedded electrode array) and sample loading (i.e. disposable cartridge) would greatly improve the user experience and reliability of sample processing. Additionally, a solid support structure that captures bacteria could more efficiently recover and concentrate the microbes in the strata containing bacteria formed during flotation.

The addition of a non-ionic surfactant (Pluronic^®^ F-68) alone to EF treatments improved concentration of *E*. *coli* 25922 and therefore improved detection rates of *E*. *coli 25922* by LAMP. The mechanism by which pluronic improves concentration of dispersed bacteria during flotation was not investigated. Interestingly, one study reports the use of surfactants to improve electro-kinetic stability of electrodes in lab-on-chip micro device by promoting smaller bubble diameters and also more rapid bubble detachment from the electrode surface [37]. While this effect was not measured directly, casual observation confirmed that fewer large bubbles formed and detached from electrodes in the presence of Pluronic. This suggests that the effect of pluronic to improve concentration of bacteria by EF extends beyond bubble-particle interactions by enabling quicker bubble detachment likely resulting in overall smaller and more uniformly sized microbubbles with smaller terminal velocities and collectively greater opportunities for collisions with suspended particles.

Surfactants modify the surface tension forces that typically attract, stress or disperse biomaterial [16]. The mechanism by which surfactants protect cells may be attributed to pluronic masking the hydrophobic properties of the cell membrane. By design, surfactants interact with bubbles resulting in local gradient changes in surface tension on the bubble surface so that a bubble will slide past a cell with lowered interactions and collision efficiency [16]. Theoretically, this should inhibit bubble-cell attachment and decrease flotation efficiency. In contrast, pluronic alone significantly improved EF concentration efficiency. Additionally, pluronic is an amphiphilic molecule that can self-assemble into microstructure micelles [38]. Surfactant micelles can encapsulate other molecules and have been used widely for the solubilization of drugs and drug delivery [39]. It is conceivable that pluronic micelles formed around detectable cell material (*i*.*e*., free DNA, lipids, cell fragments) during EF treatments. The observed increased detection rates by LAMP may be attributed to the concentration of detectable cell material otherwise not observed in corresponding EF treatments without pluronic. To investigate this hypothesis, we ran electroflotation experiments to concentrate the equivalent of 10^3^ DNA copy number/ mL, one order of magnitude below the limit of reliable detection, of *E*. *coli* under the same parameters described in “EF treatment with pluronic and chitosan”. Only 50% of the 27 LAMP assays amplified corresponding to the detection rates of the LAMP assay using purified DNA without electroflotation. From this we determined that it is unlikely that cellular DNA is concentrated during flotation and therefore only whole cells or larger detectable cell material is recovered.

Chitosan was added to electroflotation treatments to support aggregation of dispersed bacteria, which can result in substantial increase in particle (*i*.*e*., bacteria) quantity recovered. Research using chitosan as a bacterial flocculation agent for *E*.*coli* suspensions of 10^9^ CFU/mL suggests that optimal concentrations occur between 20–80 mg/g of cell dry weight depending on other factors like pH and degree of chitosan polymerization [25]. Predicting adequate chitosan concentrations based on the dry weight of cells is impractical when conducting EF on environmental samples containing unknown quantities of dispersed bacteria at low titres (<10^2^ CFU/mL). In other reports optimal chitosan or polymer concentration was found to be 10–20 μg chitosan/ billion cells [21], 25–75 g chitosan/L [40] and 20 mg chitosan/ g of *chlorella* [22]. It is generally agreed that small increases or decreases in polymer dosage can have a large effect on the stabilization of the dispersed system and significantly affect the absorption rates of the flocculant to the substrate. However, there is a lack of agreement on specific optimal chitosan concentrations reported in literature. This can be partially attributed to the challenges and complexity of quantifying properties of a dispersed colloidal system including the disagreement about the fundamental mechanism by which chitosan binds suspended solids; by direct electrostatic bridging [41] or by destabilization of charged colloidal particles by charge neutralization [42].

To our knowledge, chitosan has previously been used to flocculate large quantities of bacteria ranging from 10^7^–10^9^ CFU/mL. This is up to 7 orders of magnitude greater than the bacterial concentrations used in EF treatments in this work (10^2^−10^4^ CFU/mL). To increase the likelihood of chitosan interacting with dilute suspension of bacteria, a proportionally large dose of chitosan proportional to bacteria was added to EF treatments. For EF treatments containing ~10^2^ CFU/mL *E*. *coli*, 0.01 or 0.1 g L^-1^ was added to flocculate ~ 38,000 bacterial cells (the approximate quantity of 10^2^ CFU/mL cells dispersed in 380 mL of media).

While LAMP is generally more resistant to many common PCR inhibitors, chitosan significantly inhibited detection by LAMP. Polysaccharides commonly found in environmental samples and plant matter are well-known inhibitors of nucleic acid amplification like PCR and competitively bind to template DNA, DNA polymerases and primer binding sites, preventing the initiation of DNA amplification. By design, although not desirable, inhibitors that have aggregated during flocculation may also be concentrated during EF treatments. Many common inhibitors like polysaccharides found in environmental samples behave similarly to chitosan in that they bind to anionic particles. Our method of treating the sample with NaOH lends itself to have compounded beneficial effects and reduce inhibition from polysaccharides found in environmental samples. Diluting the sample can lower the concentration of inhibitors but would reduce the final pathogen quantities in the collected fractions. For samples containing low titers of target pathogens below the LOD of the LAMP assay this approach may be impractical.

The addition of chitosan as a flocculant to EF treated samples completely inhibited LAMP assays at concentration greater than 1 g L^-1^. At pH less than ~6.2 and below chitosan’s pKa (~pH 9.5), chitosan has a strong positive charge and will bind to negatively charged anions including template DNA inhibiting isothermal nucleic acid amplification. Our approach to prevent LAMP inhibition by chitosan was adapted from a method that successfully extracted DNA on microchips lined with chitosan coated silica beads [43]. In their system when the buffer flowing through microchannels of the device was pH 5 DNA bound to chitosan coated beads and eluted from the beads at pH 10. This method was particularly desirable because it does not require downstream DNA purification or extraction methods to remove inhibitors. The pH can be titrated in the same tube as the recovered EF sample. The crude lysis / DNA extraction step can easily be performed in portable battery-powered kettles (i.e. Cauldryn Smart Mug, St. Charles, MO, USA), or in handheld instrumentation for conducting LAMP (i.e. BioRanger^TM^ [44]) so that all methodologies in described here could be done in the field.

The EF system potentiates diverse sample preparation using a variety of sample types including irrigation water, ocean, coastal or river samples, agricultural product rinsate, drinking water, and wastewater. However, it should be noted that the electroflotation treatment conditions used to achieve reliable detection of *E*. *coli* 25922 in this study were carried out under controlled laboratory conditions and a simple buffer system. In order to apply electroflotation as a sample preparation method in POC testing scenarios on *real* agricultural or environmental samples, validation and optimization would be required for each type of sample. Important sample characteristics might include the pH of the sample, EF process settings (i.e. duration, level of turbulence and mixing, current density), surfactant and flocculant concentrations and optimization of a LAMP assay for each target pathogen. In some applications a pre-filtration step may be required if the sample contains large solid particles for example water samples containing soil or other debris and food homogenates.

A single type of environmental sample matrix can contain many different bacterial strains and/or species and therefore can host and transmit numerous types disease causing pathogens. For example, spinach products have been linked to outbreaks of *Listeria*, *Salmonella* and *E*. *coli*. A multiplexing LAMP assay that would allow simultaneous detection of all 3 bacterial strains in a single reaction tube would be ideal for agricultural samples processed by electroflotation, which indiscriminately concentrates and extracts any particles ranging from the size of 0.5 microns to 200 (*i*.*e*., bacterial pathogens) present in the sample matrix. While PCR multiplexing technology is more developed, where multiple DNA targets can be identified, LAMP technology multiplexing assays are limited and complicated to design. The more primers that are added to a single LAMP assay the greater occurrence of interference due to variances in amplification efficiencies [45]. Currently to identify pathogens in an electroflotation sample by LAMP the user would have to know what pathogen is being targeted and have primers designed for the specific pathogen. LAMP is an ideal detection technology for agricultural diagnostics and improvements in multiplexing technology that maintain sensitive and reliable detection would greatly complement POC sample preparation approaches like the electroflotation system described in this research. The realization of this technology is not far in the distant future as new methods to improve LAMP multiplexing continue to evolve including multiplexing alternatives that rely on automated parallel reactions from the sample. By replacing a specifically designed poly (T) region of the FIP primer with a target specific barcode by nicking endonuclease activity, researchers at the Nanjing University School of Medicine in China designed a four-plexed LAMP assay to detect hepatitis B virus, hepatitis C virus, human immunodeficiency virus, and *Treponema pallidum* in a single LAMP reaction tube [46]. Our lab has designed primer regions targeting spectrally unique assimilating probes so that different targets can be distinguished, potentiating application for multiplexing technologies [30].

## Supporting information

S1 File (ZIP)
